# *rac*-4-(2-Meth­oxy­phen­yl)-2,6-dimethyl­cyclo­hex-3-ene­carb­oxy­lic acid

**DOI:** 10.1107/S1600536810019732

**Published:** 2010-05-29

**Authors:** Songwen Xie, Brian P. Fowler, Sara M. Deyo, Maren Pink

**Affiliations:** a Department of Natural, Information, and Mathematical Sciences, Indiana University Kokomo, Kokomo, IN 46904–9003, USA; b Indiana University Molecular Structure Center, Indiana University, Bloomington, IN 47405–7102, USA

## Abstract

The title compound, C_16_H_20_O_3_, was synthesized to study the hydrogen-bonding inter­actions of the two enanti­omers in the solid state. Inter­molecular O—H⋯O hydrogen bonds produce centrosymmetric *R*_2_^2^(8) rings which dimerize the two chiral enanti­omers together through their carboxyl groups.

## Related literature

In similar compounds previously reported (Xie *et al.*, 2002 ▶, 2007*a* ▶, 2008*a* ▶,*b* ▶), the racemates also consist of carb­oxy­lic acid *RS* dimers. For the structure of the precursor, see: Xie *et al.* (2007*b* ▶). The chirality of the title compound is solely generated by the presence of the double bond in the cyclo­hexene ring, see: Xie *et al.* (2004 ▶). For hydrogen-bond motifs, see: Bernstein *et al.* (1995 ▶).
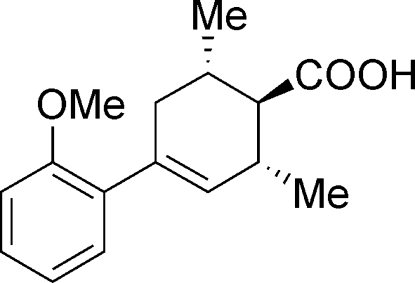


## Experimental

### 

#### Crystal data

C_16_H_20_O_3_
*M* *_r_* = 260.32
Monoclinic, 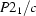

*a* = 14.2283 (9) Å
*b* = 7.1202 (5) Å
*c* = 14.9517 (10) Å
β = 106.069 (2)°
*V* = 1455.55 (17) Å^3^
*Z* = 4
Mo *K*α radiation
μ = 0.08 mm^−1^
*T* = 150 K
0.25 × 0.23 × 0.07 mm

#### Data collection

Bruker APEXII Kappa Duo diffractometer
Absorption correction: multi-scan (*SADABS*; Sheldrick, 1996 ▶) *T*_min_ = 0.980, *T*_max_ = 0.994
11165 measured reflections
2964 independent reflections
2296 reflections with *I* > 2σ(*I*)
*R*_int_ = 0.022

#### Refinement

*R*[*F* ^2^ > 2σ(*F* ^2^)] = 0.048
*wR*(*F* ^2^) = 0.138
*S* = 1.04
2964 reflections
179 parameters
H atoms treated by a mixture of independent and constrained refinement
Δρ_max_ = 0.56 e Å^−3^
Δρ_min_ = −0.34 e Å^−3^

### 

Data collection: *APEX2* (Bruker, 2007 ▶); cell refinement: *SAINT* (Bruker, 2007 ▶); data reduction: *SAINT*; program(s) used to solve structure: *SIR2004* (Burla *et al.*, 2005 ▶); program(s) used to refine structure: *SHELXL97* (Sheldrick, 2008 ▶); molecular graphics: *SHELXTL* (Sheldrick, 2008 ▶); software used to prepare material for publication: *SHELXL97*.

## Supplementary Material

Crystal structure: contains datablocks global, I. DOI: 10.1107/S1600536810019732/om2342sup1.cif

Structure factors: contains datablocks I. DOI: 10.1107/S1600536810019732/om2342Isup2.hkl

Additional supplementary materials:  crystallographic information; 3D view; checkCIF report

## Figures and Tables

**Table 1:** Hydrogen-bond geometry (Å, °)

*D*—H⋯*A*	*D*—H	H⋯*A*	*D*⋯*A*	*D*—H⋯*A*
O2—H2⋯O1^i^	0.88 (3)	1.79 (3)	2.6640 (18)	177 (3)

Symmetry code: (i) 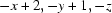.

**Fig. 1.**
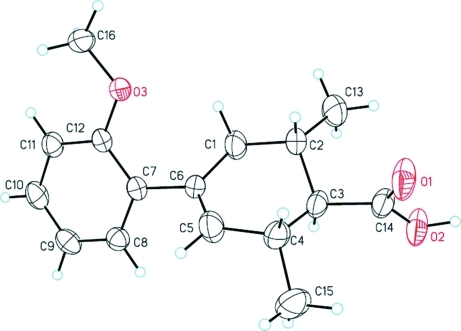
The molecular structure showing thermal ellipsoids at the 50% probability level and the atom numbering scheme.

**Fig. 2.**
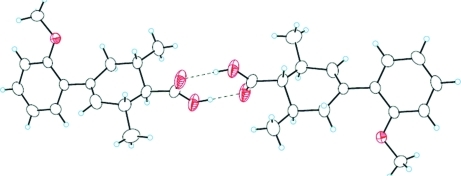
Hydrogen bonded dimer. Dashed lines represent hydrogen bonds (symmetry code: #1 -x+2,-y+3,-z).

## supplementary crystallographic information

### Comment

The title carboxylic acid was prepared to study the interaction of the two enantiomers in the solid state. We have previously reported the structure of its precursor, which is achiral and forms hydrogen-bonded dimers (Xie *et al.*, 2007b). The chirality of the title compound is solely generated by the presence of the double bond in the cyclohexene ring (Xie *et al.*, 2004). The resultant racemate is made up of carboxylic acid RS dimers (Xie *et al.*, 2002, 2007a, 2008a,b). The structure and atom numbering are shown in Fig. 1, which illustrates the half-chair conformation of the cyclohexene ring. The torsion angles involving atoms C4, C5, C6, C1, and C2 are near 0°. The carboxyl group is almost perpendicular to the cyclohexene ring with an angle of 82.2° between the O1—C14—O2—C3 plane and the C1—C6 ring. The double bond between C5—C6 is not fully conjugated with the aromatic ring as shown by the C1—C6—C5 plane to benzene ring angle of 52.6°. Unlike other previously reported *para* substituted analogs and like other previously reported *meta* substituted analogs (Xie *et al.*, 2008b), the molecule also has a chiral axis due to the *ortho* methoxy substituent on the aromatic ring.

Fig. 2 shows the hydrogen bonding scheme. Atom O2 acts as a donor in an intermolecular hydrogen bond to atom O1, producing an R22(8) ring (Bernstein *et al.*, 1995), thus creating a hydrogen- bonded dimer. There is no evidence to suggest that weak directional interactions interconnect the dimers. Hydrogen bond geometry is given in Table 1.

### Experimental

The title carboxylic acid was synthesized following asimilar method reported by Xie *et al.*, 2002. Purified compound was recrystallized from hexane- dichloromethane as colorless plates (m.p. 417-418 K).

### Refinement

All non-hydrogen atoms were refined with anisotropic displacement parameters. The hydrogen atoms not involved in hydrogen bonding were placed in ideal positions and refined as riding atoms with relative isotropic displacement parameters. H1 was freely refined.

### Crystal data

**Table:**

C_16_H_20_O_3_	*F*(000) = 560
*M**_r_* = 260.32	*D*_x_ = 1.188 Mg m^−^^3^
Monoclinic, *P*2_1_/*c*	Mo *K*α radiation, λ = 0.71073 Å
Hall symbol: -P 2ybc	Cell parameters from 4026 reflections
*a* = 14.2283 (9) Å	θ = 2.8–26.3°
*b* = 7.1202 (5) Å	µ = 0.08 mm^−^^1^
*c* = 14.9517 (10) Å	*T* = 150 K
β = 106.069 (2)°	Plate, colorless
*V* = 1455.55 (17) Å^3^	0.25 × 0.23 × 0.07 mm
*Z* = 4	

### Data collection

**Table:**

Bruker APEXII Kappa Duo diffractometer	2964 independent reflections
Radiation source: fine-focus sealed tube	2296 reflections with *I* > 2σ(*I*)
graphite	*R*_int_ = 0.022
Detector resolution: 83.33 pixels mm^-1^	θ_max_ = 26.4°, θ_min_ = 1.5°
ω and φ scans	*h* = −16→17
Absorption correction: multi-scan (*SADABS*; Sheldrick, 1996)	*k* = −7→8
*T*_min_ = 0.980, *T*_max_ = 0.994	*l* = −13→18
11165 measured reflections	

### Refinement

**Table:**

Refinement on *F*^2^	Primary atom site location: structure-invariant direct methods
Least-squares matrix: full	Secondary atom site location: difference Fourier map
*R*[*F*^2^ > 2σ(*F*^2^)] = 0.048	Hydrogen site location: inferred from neighbouring sites
*wR*(*F*^2^) = 0.138	H atoms treated by a mixture of independent and constrained refinement
*S* = 1.04	*w* = 1/[σ^2^(*F*_o_^2^) + (0.0653*P*)^2^ + 0.6215*P*] where *P* = (*F*_o_^2^ + 2*F*_c_^2^)/3
2964 reflections	(Δ/σ)_max_ = 0.001
179 parameters	Δρ_max_ = 0.56 e Å^−^^3^
0 restraints	Δρ_min_ = −0.34 e Å^−^^3^

### Special details

**Table:**

Geometry. All esds (except the esd in the dihedral angle between two l.s. planes) are estimated using the full covariance matrix. The cell esds are taken into account individually in the estimation of esds in distances, angles and torsion angles; correlations between esds in cell parameters are only used when they are defined by crystal symmetry. An approximate (isotropic) treatment of cell esds is used for estimating esds involving l.s. planes.
Refinement. Refinement of *F*^2^ against ALL reflections. The weighted *R*-factor wR and goodness of fit *S* are based on *F*^2^, conventional *R*-factors *R* are based on *F*, with *F* set to zero for negative *F*^2^. The threshold expression of *F*^2^ > σ(*F*^2^) is used only for calculating *R*-factors(gt) etc. and is not relevant to the choice of reflections for refinement. *R*-factors based on *F*^2^ are statistically about twice as large as those based on *F*, and *R*- factors based on ALL data will be even larger.

### Fractional atomic coordinates and isotropic or equivalent isotropic displacement parameters (Å^2^)

**Table:**

	*x*	*y*	*z*	*U*_iso_*/*U*_eq_	
O1	0.88885 (9)	0.4779 (2)	0.00968 (10)	0.0602 (5)	
O2	1.02302 (9)	0.3518 (2)	0.10376 (10)	0.0531 (4)	
H2O	1.051 (2)	0.411 (4)	0.0670 (18)	0.082 (8)*	
O3	0.55725 (8)	−0.14999 (17)	0.09567 (7)	0.0342 (3)	
C1	0.76051 (16)	−0.0149 (3)	0.11315 (14)	0.0478 (5)	
H1A	0.7060	−0.0803	0.0683	0.057*	
H1B	0.8079	−0.1122	0.1446	0.057*	
C2	0.81042 (14)	0.1106 (3)	0.05858 (12)	0.0418 (5)	
H2	0.7585	0.1771	0.0100	0.050*	
C3	0.87036 (12)	0.2591 (3)	0.12493 (12)	0.0366 (4)	
H3	0.9175	0.1927	0.1775	0.044*	
C4	0.80346 (14)	0.3809 (3)	0.16515 (13)	0.0409 (4)	
H4	0.7610	0.4559	0.1129	0.049*	
C5	0.73751 (16)	0.2583 (3)	0.20376 (14)	0.0479 (5)	
H5	0.7053	0.3137	0.2450	0.057*	
C6	0.72161 (11)	0.0741 (2)	0.18303 (11)	0.0300 (4)	
C7	0.66838 (11)	−0.0451 (2)	0.23497 (10)	0.0279 (4)	
C8	0.69907 (12)	−0.0459 (2)	0.33172 (11)	0.0322 (4)	
H8	0.7515	0.0337	0.3629	0.039*	
C9	0.65520 (14)	−0.1596 (2)	0.38384 (11)	0.0377 (4)	
H9	0.6772	−0.1570	0.4499	0.045*	
C10	0.57966 (14)	−0.2762 (3)	0.33929 (12)	0.0392 (4)	
H10	0.5501	−0.3557	0.3748	0.047*	
C11	0.54636 (13)	−0.2784 (2)	0.24267 (12)	0.0343 (4)	
H11	0.4942	−0.3592	0.2122	0.041*	
C12	0.58967 (11)	−0.1624 (2)	0.19099 (11)	0.0279 (4)	
C13	0.87078 (18)	−0.0056 (4)	0.00957 (17)	0.0680 (7)	
H13A	0.8984	0.0766	−0.0292	0.102*	
H13B	0.8290	−0.1004	−0.0297	0.102*	
H13C	0.9239	−0.0680	0.0560	0.102*	
C14	0.92794 (13)	0.3744 (3)	0.07423 (12)	0.0409 (5)	
C15	0.86046 (19)	0.5186 (3)	0.23835 (16)	0.0615 (6)	
H15A	0.8146	0.5943	0.2615	0.092*	
H15B	0.8997	0.6013	0.2105	0.092*	
H15C	0.9037	0.4488	0.2901	0.092*	
C16	0.47037 (14)	−0.2506 (3)	0.05064 (12)	0.0441 (5)	
H16A	0.4516	−0.2223	−0.0161	0.066*	
H16B	0.4175	−0.2129	0.0772	0.066*	
H16C	0.4822	−0.3857	0.0599	0.066*	

### Atomic displacement parameters (Å^2^)

**Table:**

	*U*^11^	*U*^22^	*U*^33^	*U*^12^	*U*^13^	*U*^23^
O1	0.0321 (7)	0.0899 (12)	0.0542 (8)	−0.0105 (7)	0.0048 (6)	0.0408 (8)
O2	0.0299 (7)	0.0756 (11)	0.0510 (8)	−0.0106 (7)	0.0066 (6)	0.0317 (7)
O3	0.0347 (6)	0.0422 (7)	0.0275 (6)	−0.0110 (5)	0.0115 (5)	−0.0011 (5)
C1	0.0570 (12)	0.0433 (11)	0.0542 (11)	−0.0197 (9)	0.0341 (10)	−0.0117 (9)
C2	0.0365 (9)	0.0554 (12)	0.0387 (9)	−0.0140 (9)	0.0190 (8)	−0.0047 (8)
C3	0.0303 (9)	0.0444 (10)	0.0336 (8)	−0.0108 (8)	0.0066 (7)	0.0099 (7)
C4	0.0461 (11)	0.0374 (10)	0.0397 (9)	−0.0125 (8)	0.0124 (8)	0.0025 (8)
C5	0.0604 (13)	0.0390 (11)	0.0554 (11)	−0.0104 (9)	0.0346 (10)	−0.0058 (9)
C6	0.0270 (8)	0.0360 (9)	0.0285 (8)	−0.0060 (7)	0.0100 (6)	−0.0011 (7)
C7	0.0277 (8)	0.0292 (8)	0.0294 (8)	0.0019 (7)	0.0121 (6)	0.0008 (6)
C8	0.0318 (9)	0.0335 (9)	0.0315 (8)	0.0020 (7)	0.0091 (7)	−0.0001 (7)
C9	0.0475 (10)	0.0398 (10)	0.0274 (8)	0.0071 (8)	0.0129 (7)	0.0056 (7)
C10	0.0515 (11)	0.0355 (9)	0.0374 (9)	−0.0013 (8)	0.0236 (8)	0.0088 (8)
C11	0.0382 (9)	0.0314 (9)	0.0374 (9)	−0.0046 (7)	0.0173 (7)	0.0010 (7)
C12	0.0301 (8)	0.0279 (8)	0.0291 (8)	0.0011 (7)	0.0138 (6)	0.0007 (6)
C13	0.0605 (14)	0.0884 (18)	0.0704 (15)	−0.0219 (13)	0.0437 (12)	−0.0283 (13)
C14	0.0299 (9)	0.0529 (11)	0.0365 (9)	−0.0122 (8)	0.0036 (7)	0.0126 (8)
C15	0.0778 (16)	0.0467 (12)	0.0576 (13)	−0.0251 (12)	0.0148 (12)	−0.0074 (10)
C16	0.0473 (11)	0.0508 (11)	0.0334 (9)	−0.0185 (9)	0.0098 (8)	−0.0066 (8)

### Geometric parameters (Å, °)

**Table:**

O1—C14	1.219 (2)	C6—C7	1.491 (2)
O2—C14	1.312 (2)	C7—C8	1.391 (2)
O2—H2O	0.88 (3)	C7—C12	1.406 (2)
O3—C12	1.3740 (19)	C8—C9	1.386 (2)
O3—C16	1.426 (2)	C8—H8	0.9500
C1—C6	1.456 (2)	C9—C10	1.376 (3)
C1—C2	1.513 (2)	C9—H9	0.9500
C1—H1A	0.9900	C10—C11	1.390 (2)
C1—H1B	0.9900	C10—H10	0.9500
C2—C13	1.519 (3)	C11—C12	1.387 (2)
C2—C3	1.537 (2)	C11—H11	0.9500
C2—H2	1.0000	C13—H13A	0.9800
C3—C14	1.504 (2)	C13—H13B	0.9800
C3—C4	1.528 (3)	C13—H13C	0.9800
C3—H3	1.0000	C15—H15A	0.9800
C4—C5	1.508 (2)	C15—H15B	0.9800
C4—C15	1.525 (3)	C15—H15C	0.9800
C4—H4	1.0000	C16—H16A	0.9800
C5—C6	1.352 (3)	C16—H16B	0.9800
C5—H5	0.9500	C16—H16C	0.9800
			
C14—O2—H2O	110.0 (18)	C9—C8—H8	119.1
C12—O3—C16	117.10 (12)	C7—C8—H8	119.1
C6—C1—C2	117.26 (16)	C10—C9—C8	119.56 (15)
C6—C1—H1A	108.0	C10—C9—H9	120.2
C2—C1—H1A	108.0	C8—C9—H9	120.2
C6—C1—H1B	108.0	C9—C10—C11	120.40 (16)
C2—C1—H1B	108.0	C9—C10—H10	119.8
H1A—C1—H1B	107.2	C11—C10—H10	119.8
C1—C2—C13	110.54 (18)	C12—C11—C10	119.74 (16)
C1—C2—C3	108.53 (14)	C12—C11—H11	120.1
C13—C2—C3	113.54 (16)	C10—C11—H11	120.1
C1—C2—H2	108.0	O3—C12—C11	122.89 (15)
C13—C2—H2	108.0	O3—C12—C7	116.24 (13)
C3—C2—H2	108.0	C11—C12—C7	120.84 (15)
C14—C3—C4	111.98 (15)	C2—C13—H13A	109.5
C14—C3—C2	109.42 (14)	C2—C13—H13B	109.5
C4—C3—C2	110.45 (14)	H13A—C13—H13B	109.5
C14—C3—H3	108.3	C2—C13—H13C	109.5
C4—C3—H3	108.3	H13A—C13—H13C	109.5
C2—C3—H3	108.3	H13B—C13—H13C	109.5
C5—C4—C15	111.17 (16)	O1—C14—O2	122.86 (16)
C5—C4—C3	110.10 (15)	O1—C14—C3	122.40 (16)
C15—C4—C3	112.43 (17)	O2—C14—C3	114.73 (15)
C5—C4—H4	107.6	C4—C15—H15A	109.5
C15—C4—H4	107.6	C4—C15—H15B	109.5
C3—C4—H4	107.6	H15A—C15—H15B	109.5
C6—C5—C4	123.77 (17)	C4—C15—H15C	109.5
C6—C5—H5	118.1	H15A—C15—H15C	109.5
C4—C5—H5	118.1	H15B—C15—H15C	109.5
C5—C6—C1	120.94 (16)	O3—C16—H16A	109.5
C5—C6—C7	120.64 (15)	O3—C16—H16B	109.5
C1—C6—C7	118.33 (15)	H16A—C16—H16B	109.5
C8—C7—C12	117.67 (14)	O3—C16—H16C	109.5
C8—C7—C6	119.05 (14)	H16A—C16—H16C	109.5
C12—C7—C6	123.25 (14)	H16B—C16—H16C	109.5
C9—C8—C7	121.77 (16)		
			
C6—C1—C2—C13	164.10 (19)	C1—C6—C7—C12	−53.0 (2)
C6—C1—C2—C3	39.0 (2)	C12—C7—C8—C9	0.9 (2)
C1—C2—C3—C14	174.85 (16)	C6—C7—C8—C9	−177.28 (15)
C13—C2—C3—C14	51.5 (2)	C7—C8—C9—C10	0.4 (3)
C1—C2—C3—C4	−61.4 (2)	C8—C9—C10—C11	−0.9 (3)
C13—C2—C3—C4	175.22 (18)	C9—C10—C11—C12	0.0 (3)
C14—C3—C4—C5	172.34 (15)	C16—O3—C12—C11	5.2 (2)
C2—C3—C4—C5	50.1 (2)	C16—O3—C12—C7	−172.68 (15)
C14—C3—C4—C15	−63.1 (2)	C10—C11—C12—O3	−176.46 (16)
C2—C3—C4—C15	174.67 (15)	C10—C11—C12—C7	1.4 (2)
C15—C4—C5—C6	−141.8 (2)	C8—C7—C12—O3	176.16 (14)
C3—C4—C5—C6	−16.6 (3)	C6—C7—C12—O3	−5.7 (2)
C4—C5—C6—C1	−6.3 (3)	C8—C7—C12—C11	−1.8 (2)
C4—C5—C6—C7	170.29 (17)	C6—C7—C12—C11	176.29 (15)
C2—C1—C6—C5	−5.8 (3)	C4—C3—C14—O1	−56.8 (3)
C2—C1—C6—C7	177.46 (16)	C2—C3—C14—O1	66.0 (3)
C5—C6—C7—C8	−51.6 (2)	C4—C3—C14—O2	124.50 (18)
C1—C6—C7—C8	125.06 (18)	C2—C3—C14—O2	−112.68 (19)
C5—C6—C7—C12	130.28 (19)		

### Hydrogen-bond geometry (Å, °)

**Table:**

*D*—H···*A*	*D*—H	H···*A*	*D*···*A*	*D*—H···*A*
O2—H2O···O1^i^	0.88 (3)	1.79 (3)	2.6640 (18)	177 (3)

Symmetry codes: (i) −*x*+2, −*y*+1, −*z*.
